# Passengers’ seat vibration exposure on turboprop aircraft flights

**DOI:** 10.1177/10519815251319224

**Published:** 2025-02-25

**Authors:** Neil J Mansfield, Peter Vink, Gerbera Vledder, Xinhe Yao, Britta Herbig, Anna S Reichherzer, Michael Bellmann

**Affiliations:** 1 Nottingham Trent University, Nottingham, UK; 2 VHP Human Performance, The Hague, The Netherlands; 3 Faculty of Industrial Design Engineering, Delft University of Technology, Delft, The Netherlands; 4 Ludwig-Maximilians-Universität München, Munich, Germany; 5 Institut für technische und angewandte Physik GmbH, Oldenburg, Germany

**Keywords:** vibration, aviation, aircraft, comfort, ergonomics, human factors

## Abstract

**Background:**

Turboprop aircraft offer the possibility of lower emissions for regional travel in comparison to jets. Future low-carbon aircraft concepts include propeller-generated thrust powered from fuel cells, hydrogen, biofuel, battery or hybrid power. The noise and vibration experienced in a turboprop cabin is different to that experienced in a jet, with signals characterised by tonal components related to the propeller blade pass frequency. These components have been associated with noise and vibration discomfort. There are few published studies of aircraft cabin vibration measured on the seat surface according to ISO2631-1; none report data for the whole flight.

**Objective:**

The objective was to measure and evaluate the vibration experienced by passengers for complete turboprop flights and compare vibration data with standards associated with vibration comfort.

**Methods:**

Vibration data was measured on the surface of three occupied seats during two turboprop aircraft flights. Measurements were made on full flights, and covered the entire duration from gate-to-gate.

**Results:**

Data showed that the vibration is highly tonal, and is affected by position and flight phase. Frequency-weighted vibration showed magnitudes below thresholds for health risk. The highest magnitudes of vibration occurred at the blade pass frequency and its harmonics. These frequencies are rejected by standard comfort assessment methods that use frequency weightings.

**Conclusions:**

Whole-body vibration exposure in the turboprop tested in this study did not approach health risk thresholds using ISO2631-1. Analysis of the vibrational comfort requires use of band-limited vibration assessment methods to include the dominant vibration components in analysis.

## Introduction

Demand for passenger air travel is expected to continue to grow despite the shift in working patterns and communication norms triggered by the global pandemic.^1^ Regional passenger transport usually occurs on single aisle aircraft, including those powered by turbojet engines (‘jets’ such as Boeing 737 or Airbus A320 series) or turboprops (such as ATR 42/72 or Bombardier Q400/Dash 8). Turboprops generate power through rotation of the propeller, the wake from which interacts with the wing resulting in the tonal component related to the blade pass frequency. Future aircraft may use alternative power sources such as electric or hybrid systems also likely to use propellers. Future personal air transport (‘air-taxi’) concepts currently use propeller derived thrust.^2^

Passengers perceive propeller aircraft as being uncomfortable due the noise and vibration.^3^ This is more a problem in turboprops than jet aircraft where noise and vibration are important, but lower ranked in terms of discomfort.^4^ A review of the literature showed that there have been very few published studies of the vibration experienced by passengers in aircraft cabins.^5^ Studies rarely conducted vibration measurements according to ISO2631-1 or measured the entire flight from gate-to-gate. Where data has been reported it has been focused on pilots, military aircraft, or just short parts of a flight. Data measured on the aircraft structure has shown that there are tonal peaks at frequencies above 80 Hz for both jets and turboprops.^6^ These frequencies are heavily attenuated by the standard method for assessment of human response to vibration, ISO2631-1;^7^ for example, vibration at 100 Hz 98% and 91% of the signal is removed in the horizonal and vertical directions respectively.

Previous research has shown that for low magnitudes of vibration the frequency weightings used in ISO2631-1 are better matched to reported comfort and perception at low frequencies (<20 Hz) than at high frequencies.^8^ These findings imply that high frequency vibration is much more important to passengers than the frequency weightings indicate because the magnitudes are low. The weightings perform much better at predicting comfort for higher magnitudes of vibration such as occurs in industrial exposures to vibration.^9^

Despite the potential growth of propeller aircraft and the large population of users, there remains little published data describing the vibration experienced on the seat of passenger aircraft. This paper reports vibration data measured on the surface of seats in an ATR72 turboprop aircraft. Two fully occupied test flights were conducted as part of the ComfDemo project.^10^

## Methods

Data was collected on two flights of 70 min duration reaching a cruising altitude of 17,000 feet. Data was also collected during the taxi from the gate and on the return to the gate. The aircraft was an ATR72-500 with a capacity of 60 passengers. It was full and specially chartered for the test flights. The aircraft was configured in a 2 × 2 layout with 35” seat pitch. All passengers gave signed consent for the data collection; the study was approved by (1) the Human Research Ethical Committee (HREC) of Delft University of Technology under file number 1823; (2) the Ethics Committee at the Faculty of Medicine, Ludwig-Maximilians-University, Munich, under ID 21-1010. Personal data from particpants is not presented in this paper.

**Figure 1.**
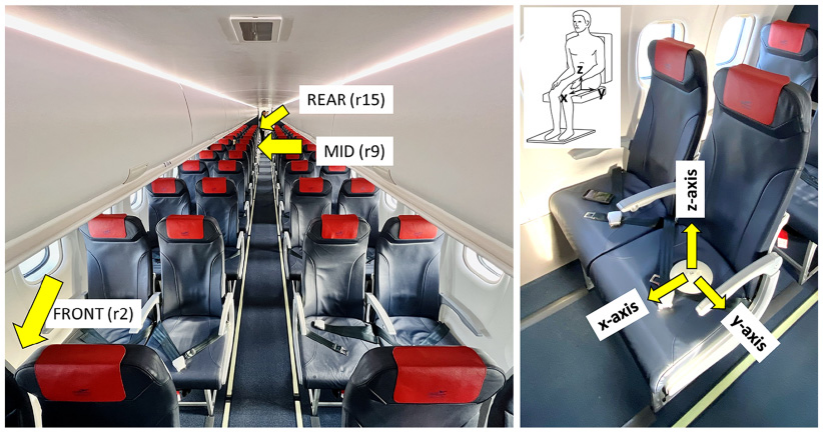
Position of accelerometers in the ATR aircraft and illustration of the basi-centric axes of the human body considered for evaluation of WBV in accordance with ISO2631-1.

Whole body vibration was measured on the surface of three occupied seats in the aircraft cabin in accordance with ISO2631-1. Seat occupants were briefed about the purpose of the vibration measurements and instructed to not adjust the seat pad. The seats were located on different rows representing front (p1), middle (p2) and rear (p3) positions. Measurements were made using Axivity AX3 triaxial accelerometer and data loggers which were mounted inside a seat pad (Figure 1). Calibration was checked before each flight (ATR01 and ATR02). The AX3s were configured to sample at 800 Hz with a range of 2 g. Data was collected simultaneously in x-, y-, and z-axes as marked on the accelerometer (Figure 1). Seat occupancy was measured using a thermal sensor that detected the presence of a human body. Measurements were conducted for the full duration of the flights. Data segments were extracted from the measurements for full analysis. Each segment was checked to ensure that the occupant did not leave the seat during the sample.

The data segments were selected to represent the different flight phases. ‘Taxi’ was selected as the time from leaving the gate to when the aircraft entered the runway. ‘Runway/Take-off’ was selected as the time from accelerating on the runway to when the power was cut back immediately after take-off. ‘Climb’ was selected as the period of the climb before cruise altitude was achieved. ‘Cruise’ was selected as time maintained at cruising altitude obtained from flight data. ‘Approach/Landing’ included the final part of approach and the time on the runway while the aircraft decelerated. Five-minute data samples were extracted for Taxi, Climb, Cruise and Approach/Landing. Runway/Take-off duration lasted approximately 40 s and this was used as the test sample.

Data analysis was conducted in MATLAB and included frequency weighted signals, in accordance with ISO2631-1, and the use of unweighted band limited signals. Frequency weighted signals in each direction (x-, y-, z-axis) were considered individually and also combined using the root-sum-of-squares method, after scaling x- and y-axis data by 1.4 in accordance with ISO 2631-1. Weightings of Wk and Wd were used for vertical and horizontal vibration respectively. This method is recommended for comfort assessment as was the focus of this study.

## Results

**Figure 2.**
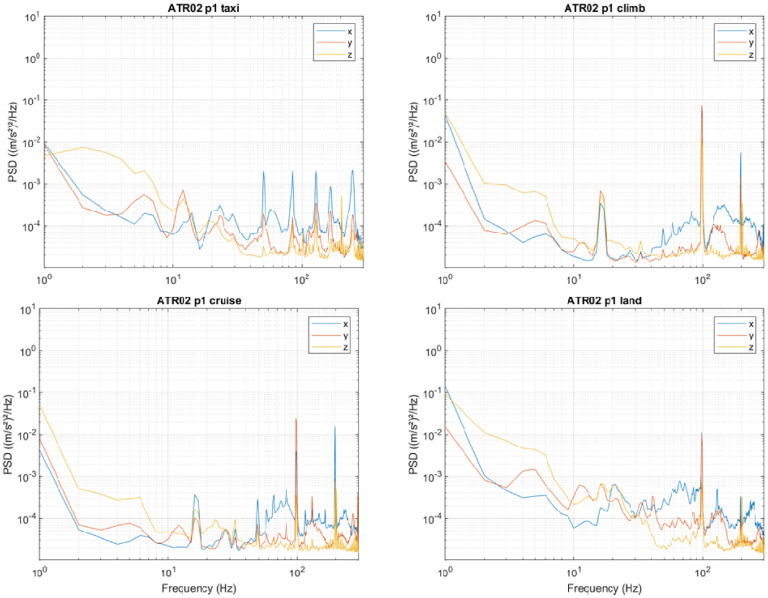
Power Spectral Density of vibration measured on the surface of an aircraft seat during four flight phases; flight 2.

For most phases of the flight, the vibration was dominated by tonal components (Figure 2). The first peak during the climb and cruise phase occurred at 16.5 Hz, with additional components at 35, 49 and 98 Hz. During landing the 16.5 Hz component was reduced but higher frequency harmonics were still present. During landing lower frequency components were also increased.

**Figure 3.**
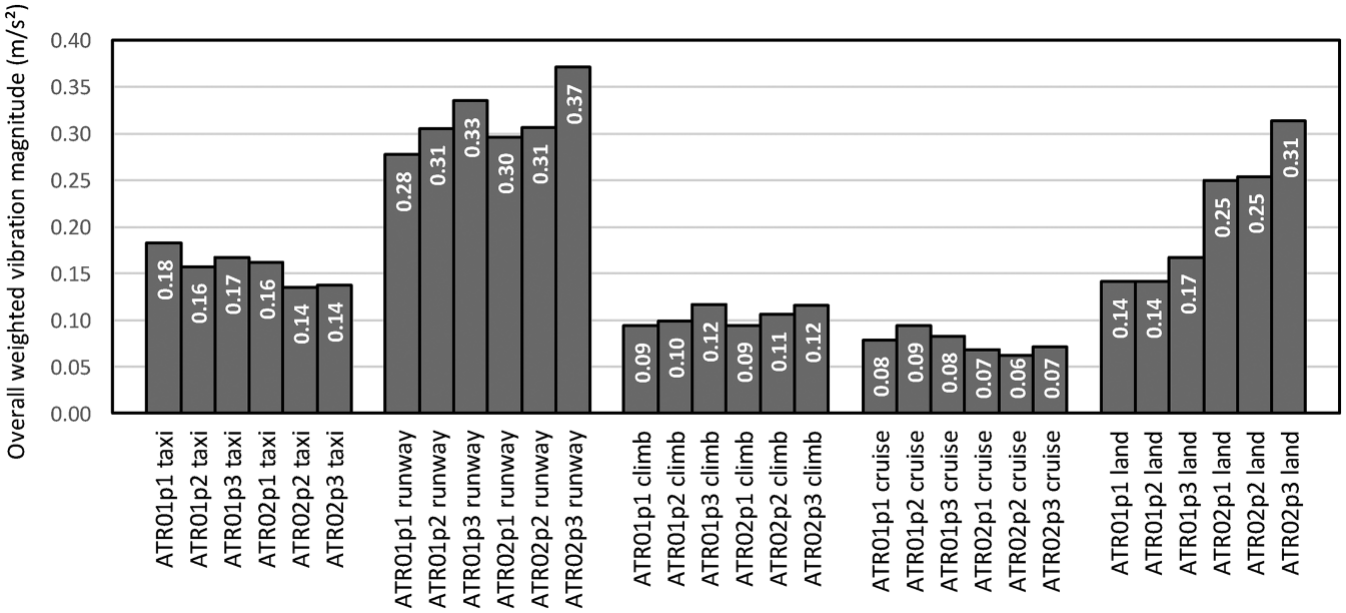
Overall r.s.s. weighted vibration magnitudes during two flights (ATR01 and ATR02), five flight phases, and three seating positions (p1, p2 and p3).

**Figure 4.**
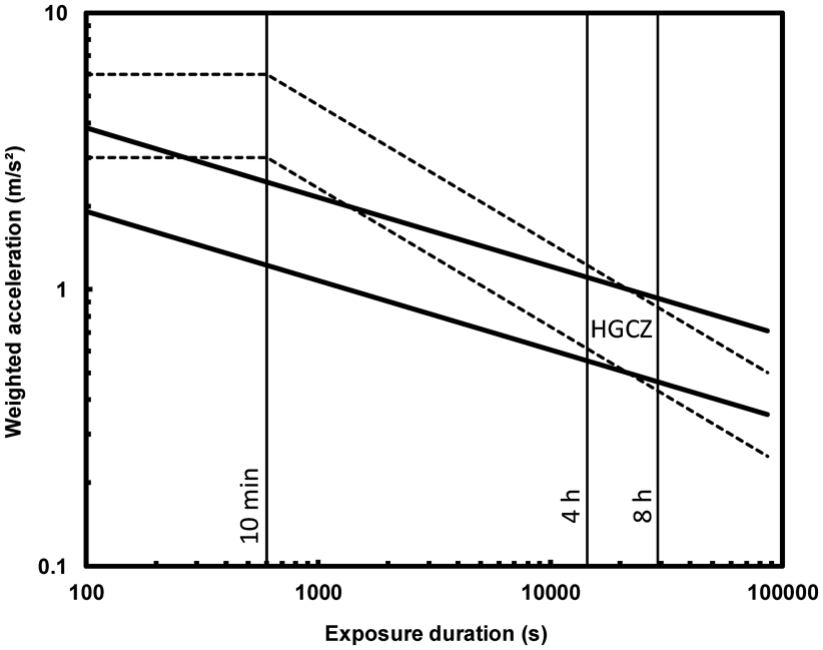
Health guidance caution zones as defined in ISO 2631-1. Dotted lines show zone defined using r.m.s.; solid lines show zone defined using VDV. Between 4 and 8 h the zone is similar for both calculation methods.

Frequency weighted vibration data showed that the highest vibration magnitude was experienced during runway and takeoff and during landing (Figure 3). Taxi exposed passengers to more frequency weighted vibration than the climb or cruise phase. For most flight phases there was more vibration experienced towards the rear of the aircraft (p3) than towards the front of the aircraft (p1). Vibration magnitudes during the cruise phase were less than 0.1 m/s^2^ r.m.s. During the taxi phase more vibration was experienced towards the front of the aircraft. More vibration was experienced during landing for the second flight in comparison to the first flight. The frequency-weighted magnitudes were below the Health Guidance Caution Zone in ISO261-1 for each axis, indicating that vibration exposure risk is low (Figure 4).

Frequency weighted vibration in the y-axis increased towards the rear of the aircraft during runway and landing flight phases (Table 1). For each phase and each flight, frequency-weighted vibration was greatest in the vertical direction.

**Table 1.** Frequency weighted vibration magnitudes measured on the surface of three seats (p1, p2, p3) during two turboprop flights (ATR01 and ATR02). Frequency weighting Wk was used for vertical (z) vibration and Wd for horizontal vibration (x and y). Axis scaling factors have not been applied in these data.

Flight phase and axis of measurement	ATR01p1 (m/s² r.m.s.)	ATR01p2 (m/s² r.m.s.)	ATR01p3 (m/s² r.m.s.)	ATR02p1 (m/s² r.m.s.)	ATR02p2 (m/s² r.m.s.)	ATR02p3 (m/s² r.m.s.)
taxi x	0.045	0.040	0.045	0.048	0.044	0.042
taxi y	0.056	0.037	0.068	0.046	0.036	0.048
taxi z	0.152	0.138	0.122	0.132	0.109	0.105
runway x	0.074	0.077	0.074	0.094	0.095	0.104
runway y	0.055	0.087	0.120	0.067	0.098	0.138
runway z	0.245	0.258	0.270	0.249	0.240	0.281
climb x	0.029	0.040	0.024	0.036	0.041	0.034
climb y	0.025	0.041	0.029	0.021	0.037	0.038
climb z	0.077	0.058	0.103	0.074	0.073	0.091
cruise x	0.019	0.023	0.015	0.019	0.017	0.016
cruise y	0.022	0.037	0.039	0.024	0.019	0.027
cruise z	0.068	0.072	0.059	0.053	0.050	0.056
land x	0.045	0.044	0.040	0.084	0.078	0.077
land y	0.050	0.065	0.077	0.078	0.108	0.154
land z	0.105	0.089	0.115	0.191	0.172	0.200

**Figure 5.**
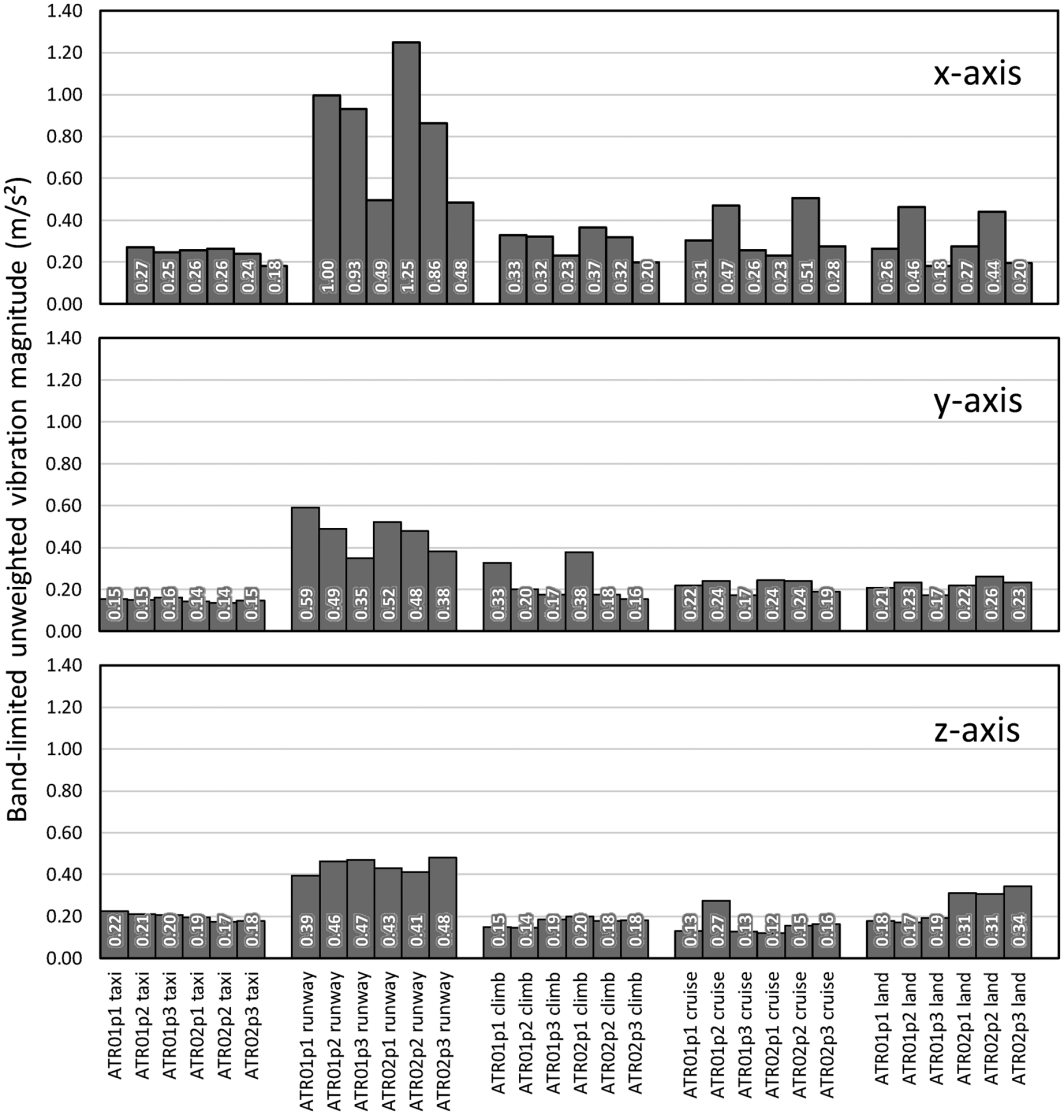
Band-limited (0.4 to 300 Hz) unweighted vibration magnitudes during two flights (ATR01 and ATR02), five flight phases, and three seating positions (p1, p2 and p3).

Vibration data were bandlimited in the frequency range of 0.4 to 300 Hz in order to include vibration data at higher frequencies than those emphasized by the International Standard frequency weightings. For most flight phases the highest magnitudes of vibration were measured in the x-axis (fore-and-aft, Figure 5). Z-axis vibration (vertical) was higher for flight 2 in comparison to flight 1 for two of the three measurement positions. The highest magnitude of vibration was observed for the runway phase of the flight, with more vibration towards the front of the aircraft than the rear.

## Discussion

The tonal nature of the vibration confirmed the findings of previously reported measurements of a different type of turboprop that measured on the aircraft structure rather than on the surface of the seat.^6^ The main engine rotation speed for the ATR is rated at 1000 rpm, which corresponds to the 16.5 Hz peak observed in the data. The first harmonic corresponds to the blade-pass frequency. This aircraft used 6-bladed propellors and therefore the blade pass frequency would be expected to occur at 100 Hz.

During approach and landing there is an increased component of air turbulence and aerodynamic effects. This was observed in the measurements that showed an increase in low frequency vibration components for the approach and landing in comparison to cruise phase. The two flights occurred on the same day but the weather became poorer for the second flight with increased cloud and rain. This increased the turbulence on approach as can be observed in the data from the two flights.

For flight phases there is more vibration at the back of the aircraft than at the front. However, when it was on the ground this trend was reversed. It is likely that this was caused by the difference in the aircraft dynamics when supported on the undercarriage and suspension in comparison to when load is taken by the wings.

The absolute frequency-weighted magnitudes of vibration experienced in the aircraft would be considered ‘not uncomfortable’ in road passenger transport according to ISO 2631-1's guidance. However the standard states that reactions are highly dependent on context, and also notes that in some environments (e.g., buildings) occupants are likely to complain even if magnitudes are only just above perception thresholds of approximately 0.015 m/s^2^ peak weighted. The lowest vibration magnitudes measured in this study were about 4 times higher than perception thresholds, and most measurements were higher still. According to ISO 2631-1, horizontal axes can optionally be scaled by a factor of 1.4 for comfort evaluation if measurements are only taken at the seat surface, as in this case. When scaled, the y-axis magnitude at the rear of the aircraft was similar to vertical for the landing phase, indicating the importance of lateral motion at specific times during the flight (Table 1).

**Figure 6.**
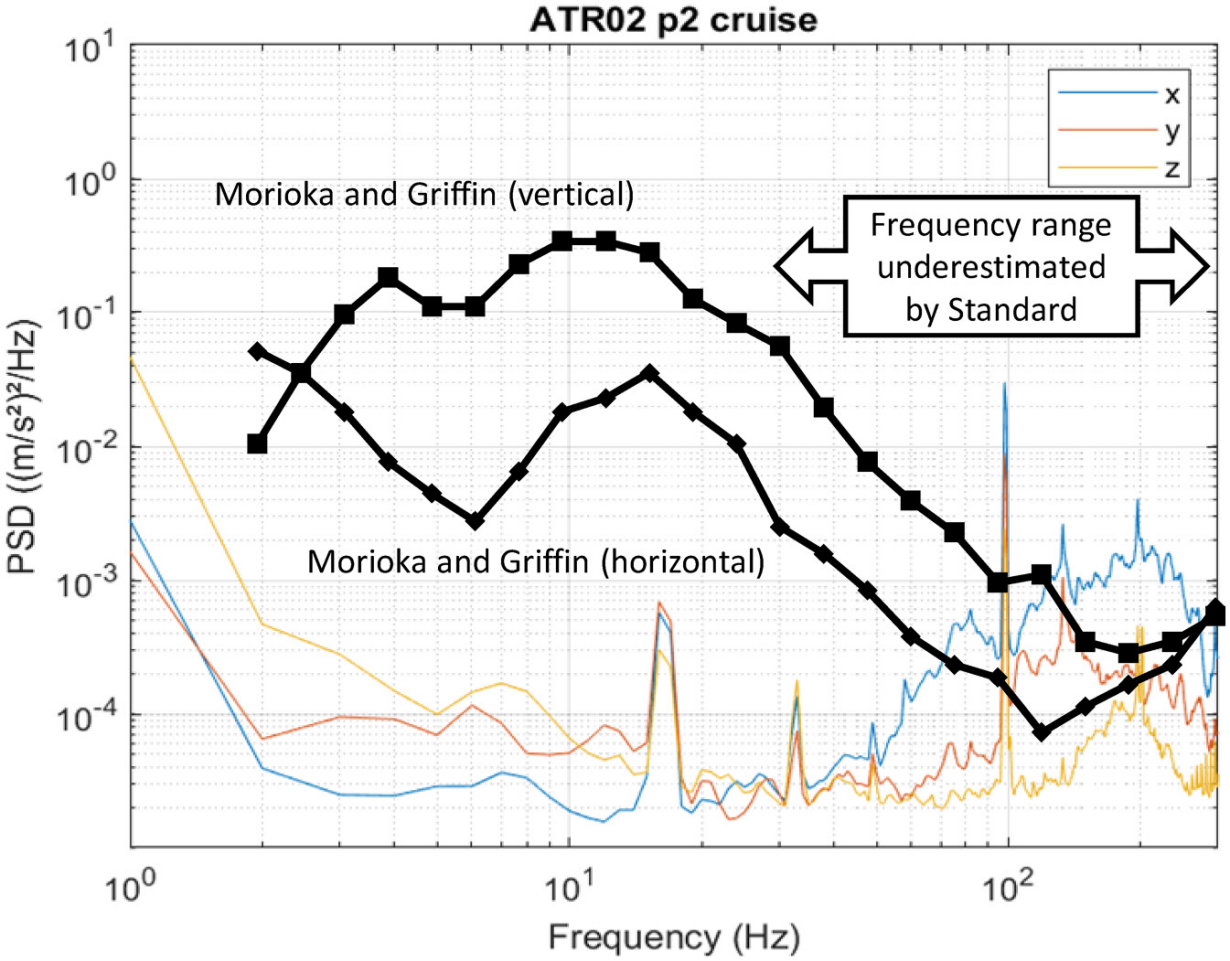
Comparison of measured vibration spectrum with previously published data showing the frequencies at which participants are more sensitive than indicated by ISO 2631-1. (Morioka and Griffin, 2008. Lower value indicates more sensitive; data are non-dimensional and do not directly indicate vibration thresholds).

Morioka and Griffin^8^ demonstrated that at low magnitudes of vibration perception thresholds map poorly to the International Standard frequency weightings. Using laboratory experiments they showed that at frequencies above about 20 Hz, people are more sensitive to whole-body vibration than would be implied by standard assessments. This might not be impactful for many environments where lower frequencies dominate but in this study it is shown that in a turboprop there is substantial vibration at high frequencies, especially at the blade-pass frequency of 100 Hz. This corresponds to the frequency at which the human response to vibration is most under-represented by the Standard (Figure 6).

Analysis of band-limited unweighted vibration allows for the inclusion of high-frequency components in comfort assessments, that would otherwise be removed from the data. Frequency analysis of the data (Figures 2 and 5) shows that components are present to 300 Hz. These should be included in data analysis for the purposes of comfort assessment.

## Limitations

This study measured data on two flights of the same aircraft on the same day. To generalize the results additional measurements should be made on other flights in different aircraft. This study did not consider vibration at other input points (e.g., armrests, feet) and these should also be considered if seeking to have a full understanding of the passenger environment.

## Conclusions

Vibration magnitudes in turboprop aircraft are low when compared to industrial vibration exposure but are a contributing factor to discomfort for passengers. The most vibration is experienced during take-off and landing phases of the flight. Vertical vibration tends to dominate the frequency weighted vibration exposure, but horizontal vibration is also an important contributor and should not be neglected.

It is recommended that evaluations of comfort in aircraft cabins includes consideration of the vibration at the seat. It is recommended that vibration is assessed using both standardized methods with frequency weightings (for comparison with previous data) and also bandlimited vibration up to 300 Hz. The contribution of high frequencies of vibration to perceptions of comfort is poorly understood and requires further study in the context of aircraft cabin environments. This is of particular importance with the anticipated growth of passenger air transportation with propeller-based power systems.
